# Understanding the Leakage Mechanisms and Breakdown Limits of Vertical GaN-on-Si p^+^n^−^n Diodes: The Road to Reliable Vertical MOSFETs

**DOI:** 10.3390/mi12040445

**Published:** 2021-04-16

**Authors:** Kalparupa Mukherjee, Carlo De Santi, Matteo Buffolo, Matteo Borga, Shuzhen You, Karen Geens, Benoit Bakeroot, Stefaan Decoutere, Andrea Gerosa, Gaudenzio Meneghesso, Enrico Zanoni, Matteo Meneghini

**Affiliations:** 1 Department of Information Engineering, University of Padua, 35131 Padova, Italy; desantic@dei.unipd.it (C.D.S.); matteo.buffolo@dei.unipd.it (M.B.); gerosa@dei.unipd.it (A.G.); gauss@dei.unipd.it (G.M.); zanoni@dei.unipd.it (E.Z.); menego@dei.unipd.it (M.M.); 2 Imec, Kapeldreef 75, 3001 Leuven, Belgium; matteo.borga@imec.be (M.B.); shuzhen.you@imec.be (S.Y.); karen.geens@imec.be (K.G.); stefaan.decoutere@imec.be (S.D.); 3 CMST IMEC/UGent, 9052 Ghent, Belgium; benoit.bakeroot@imec.be

**Keywords:** semi-vertical, vertical, GaN, pn diodes, leakage modeling, device modeling, TCAD

## Abstract

This work investigates p^+^n^−^n GaN-on-Si vertical structures, through dedicated measurements and TCAD simulations, with the ultimate goal of identifying possible strategies for leakage and breakdown optimization. First, the dominant leakage processes were identified through temperature-dependent current–voltage characterization. Second, the breakdown voltage of the diodes was modelled through TCAD simulations based on the incomplete ionization of Mg in the p^+^ GaN layer. Finally, the developed simulation model was utilized to estimate the impact of varying the p-doping concentration on the design of breakdown voltage; while high p-doped structures are limited by the critical electric field at the interface, low p-doping designs need to contend with possible depletion of the entire p-GaN region and the consequent punch-through. A trade-off on the value of p-doping therefore exists to optimize the breakdown.

## 1. Introduction

Wide bandgap semiconductors (WBGs) are being widely advocated to meet the demands for higher efficiency, robustness, and power handling capabilities [1,2,3,4,5,6,7,8,9,10,11,12,13,14,15,16], as Si is approaching its limits in market sectors where high efficiency and high power are required, such as transportation, aerospace, and power conversion systems. The contenders include SiC [13,14], GaN, β-Ga_2_O_3_ [15], diamond [11,12], and AlN [16] in order of increasing bandgap and thus breakdown field. So far, only GaN and SiC have progressed from research-level devices to commercially available technologies, whereas β-Ga_2_O_3_, diamond, and AlN prototypes, while highly promising, have not yet reached the maturity for high level production.

Currently, SiC-based devices are dominating the WBG market for power applications requiring >1200 V, voltages that are not yet reached by commercial lateral GaN devices. However, GaN is superior to SiC in overall material properties (breakdown field, mobility, saturation velocity), making it the stronger choice for most applications. To further enhance the performance of GaN transistors, the focus of research is shifting from lateral to vertical architectures, which circumvent the breakdown limitations, surface trapping, and other challenges inherent to the lateral topology.

The adoption of GaN-based vertical diodes and transistors [1,2,3,4,5,6,7] is highly advantageous to high-speed and high-power electronics applications, presenting low R_on_ and higher breakdown robustness in addition to improved thermal performance. Preliminary demonstrators of quasi-vertical or vertical diode structures [1,2,3,4,5,6,7,8,9,10] have already presented good performance metrics, handling the associated epitaxial challenges in various ways.

Within the GaN power field, emerging GaN-on-Si vertical technologies have the competitive advantage. However, owing to the lattice mismatch between GaN and Si, the epitaxial thicknesses and doping of the vertical stack require careful optimization to achieve high breakdown voltages. For well-designed structures, the drift region becomes especially relevant because it dominantly controls the reverse breakdown capability. Currently, the wide use of GaN-on-Si vertical devices is being impeded by the lack of understanding of the leakage mechanisms and breakdown of the vertical stack. Leakage paths along the passivation, drift, and other transition layers, and through the Si substrate can critically affect the reliability of GaN-on-Si diodes. Reverse leakage conduction can arise from electrode limited, surface limited, or bulk limited conduction mechanisms [5,7,17].

Hence, the study and identification of vertical leakage conduction and doping constraints to optimize breakdown voltage are major research concerns in the design of vertical pn GaN diodes and FETs [5,6,7]. This analysis is different from studies of leakage in lateral GaN devices. In lateral GaN transistors, the buffer is typically isolating and compensated with carbon; in vertical GaN devices, most of the stack is constituted by a drift region that is not intentionally doped and which needs to be highly conductive. To investigate the leakage and breakdown, technology computer aided design (TCAD) simulations provide cost-effective and versatile perspectives towards the optimization and design of these devices. In this work, we use a combination of TCAD simulations and experimental measurements to understand how to overcome the breakdown and leakage limits of GaN-on Si vertical devices, and to provide general design rules.

Within this work, Section 2 presents the structural details of the investigated quasi-vertical GaN-on-Si diodes. Section 3 is divided into two parts: Section 3.1 presents the modeling of the reverse leakage current of the tested devices, to identify the dominant mechanism for low and high voltage ranges, while Section 3.2 uses a simplified TCAD model to understand how the nature of breakdown changes as a function of the chosen doping level within the p^+^ body. Section 4 reviews the main results and concludes the work.

## 2. Materials and Methods

**Figure 1:**
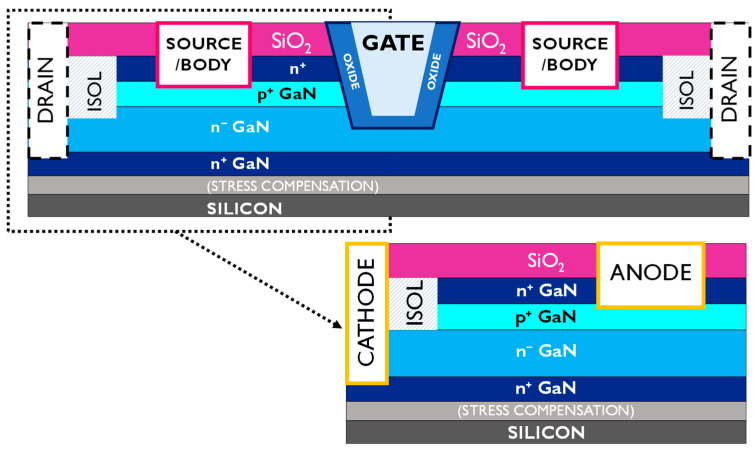
Schematic of the p^+^n^−^ diode test vehicle structures, the fundamental unit of a complete semi-vertical MOSFET.

The p^+^n diode is the core of the epitaxy of semi-vertical/vertical trench-MOSFETs [8,9,10]; hence, understanding its breakdown limits is fundamental for the development of robust vertical GaN transistors. The samples in this study are test vehicles for investigation into the p^+^n^−^n GaN-on-Si semi-vertical diode configuration (Figure 1), enabling the dedicated characterization of diode properties and doping effects.

Fabricated on a 200 mm Si substrate, the diodes are based on an Mg-doped p^+^ GaN body, where N_A_ = 6 × 10^19^ cm^−3^, and a lightly doped n^−^ drift layer. The cathode is defined at the buried n^+^ layer below the n-drift region. The n^+^ layers have a doping of 5 × 10^18^ cm^−3^, while the n^−^drift layer has a doping of N_D_ = 4 × 10^16^ cm^−3^. The parameters have been summarized in Table 1. For the p^+^ GaN, the value of N_A_ = 6 × 10^19^ cm^−3^ represents the test structures under test. However, the simulated p-doping levels have been varied to discuss the impacts of choosing low versus high p-doping values.

## 3. Results and Discussion

### 3.1. Physical Origin of Leakage Current

The conduction in the depletion region of a reverse biased p^+^n junction can be considered analogous to leakage through a dielectric subjected to high fields [5,7,18]. The off-state leakage mechanisms can then be categorized with respect to (a) properties of the metal-dielectric (semiconductor) contact—referred to as electrode-limited conduction mechanisms, or (b) the properties of the dielectric, and thus the existing trap levels—referred to as bulk-limited conduction mechanisms. Both kinds of processes might be simultaneously applicable; however, electrode-limited mechanisms such as thermionic emission, Schottky emission, and direct or F–N tunneling should not be the limiting factors in well-designed vertical diodes with the peak field located deeper at the p^+^n junction.

**Figure 2:**
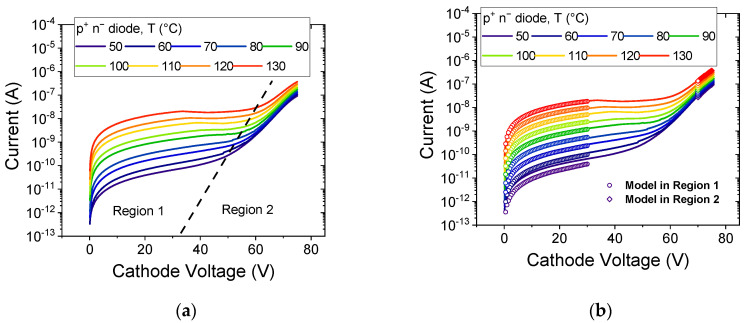
(**a**) Reverse leakage of the p^+^n^−^n diodes measured for T = 50 °C to 130 °C. Two distinct regions can be identified; (**b**) illustrates the fit to the models used to describe the leakage evolutions in the two regions.

As such, the dominant mechanisms are usually bulk-limited, such as Poole–Frenkel emission, space charge limited conduction (SCLC), and variable range hopping (VRH), among others. To identify the vertical leakage mechanisms of the studied p^+^n^−^n diodes, the temperature (T) dependence of the reverse biased diode characteristic is measured, as illustrated in Figure 2a.

The curve exhibits two distinct regions (Region 1 and Region 2), with a notable second rise in slope for voltages higher than 40–60 V (depending on temperature). Each region has been modelled separately, as illustrated in Figure 2b, and described as follows.

**Figure 3:**
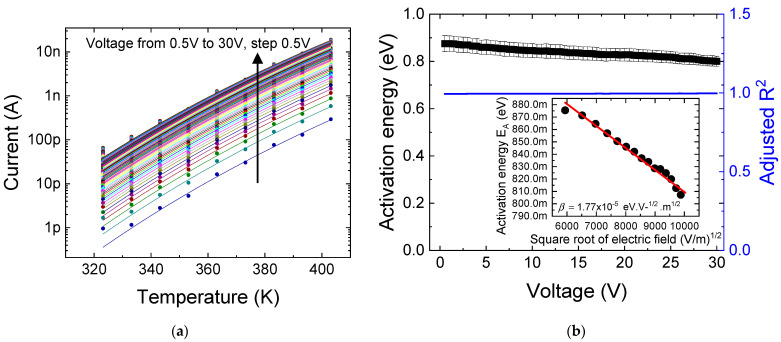
(**a**) Modeling reverse leakage diode behavior using Coulombic potential well model from 0 V to −30 V (Region 1) for T = 50 °C (323 K) to 130 °C (403 K); (**b**) extrapolated trap activation energy of 0.85 eV. The R^2^ of the fit is near one in the entire analyzed voltage range, confirming the good quality of the fit. (inset) Lowering in E_A_ showing F^−1/2^ dependence.

The observed rapid rise in leakage with temperature implies a strong thermally activated process, which is modelled in Figure 3a using the following equation:

(1) ITE=AT2exp(−EAkBT),

This equation describes the temperature dependence of the current conduction originated by thermionic emissions from Coulombic traps [6,19]. Here, *A* is a constant defining the almost vertical shift of the curve, $E_{A}$ is the thermal activation energy defining the slope of the curve, *k_B_* is the Boltzmann constant, and *T* is the temperature. An activation energy of ≈0.85 eV is extracted from the slope in Figure 3b, indicating a possible role of carbon acceptors in the leakage process [20,21].

**Figure 4:**
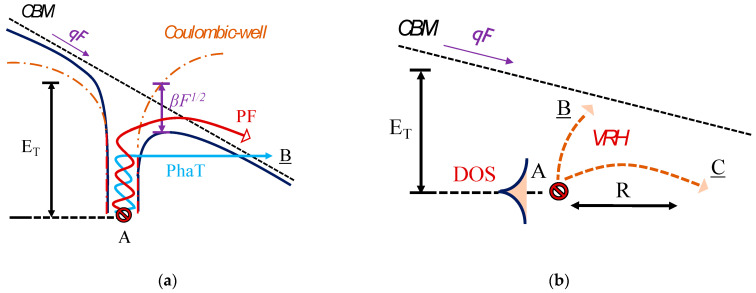
Schematic representation of the leakage processes: (**a**) Poole–Frenkel (PF) emission (used to model Region 1 in Figure 2); (**b**) variable range hopping (used to model Region 2 in Figure 2).

The corresponding conduction mechanism is described in Figure 4a. At low field F, the potential near traps can be assumed to be Coulombic, while at higher fields, the potential is deformed. Depending on the nature of the deformation, charge emission from an occupied primary trap state located at an energy of *E_T_* from the conduction band minimum (CBM) (labelled A in Figure 4) can be strengthened through different conduction mechanisms. A higher temperature can lead to phonon assisted tunneling processes (contribution labelled PhaT in Figure 4a). With increasing temperature, the overall thermal energy of the trapped electron is higher, leading to a thinner barrier for carrier tunneling, as illustrated in the transition from A to B in blue in Figure 4a. However, under high fields, Poole–Frenkel lowering of the barrier height becomes relevant. A lower effective barrier can be directly overcome by the carriers by thermionic emission (contribution labelled PF in Figure 4a), as illustrated in the transition from A to B in red in Figure 4a. The Poole–Frenkel effect thus results in a change in the emission rate $e_{n}$ [7,19,22,23] as follows:

(2) en∝exp(−ET−βF12kBT).

This process facilitates emissions from trap centers at high fields (the Poole–Frenkel coefficient *β* quantifies the lowering in the barrier = *β*√*F*). As presented in the inset of Figure 4b, the field dependence of the extracted trap level at 0.85 eV is found to follow this behavior. The peak electric field values are taken from corresponding numerical simulations in the considered voltage range (described in detail in Section 3.2).

**Figure 5:**
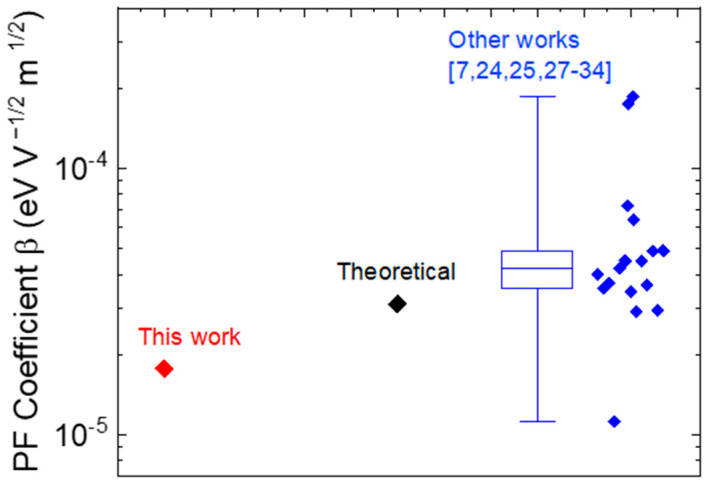
Comparison of the Poole–Frenkel coefficient (β) ≈ 10^−5^ eV V^−1/2^ m^1/2^ obtained from different works on GaN-based systems [7,24,25,27,28,29,30,31,32,33,34].

Theoretically, *β* can written as β=Zq3πε [7,24,25], where *Z* represents the charge on the Coulomb center (ionization state of the trap), and ε is the permittivity of GaN. The extracted *β* = 1.77 × 10^−5^ eV V^−1/2^ m^1/2^ in our measurements is close to the theoretical value (≈3.1 × 10^−5^ eV V^−1/2^ m^1/2^) considering a relative high-frequency GaN permittivity of 5.8, and *Z* = 1 for the carbon acceptor. Considering a simplified β=qπε [26], theoretical values are close to 3.2 × 10^−5^ eV V^−1/2^ m^1/2^ [27,28,29]. Depending on the growth conditions in different GaN-based works, β ≈ 10^−5^ eV V^−1/2^ m^1/2^ are generally reported [7,24,25,27,28,29,30,31,32,33,34], as summarized in Figure 5.

For the second region of the leakage curves, the variable range hopping [6,7,35] model, which describes the current associated with the hopping of electrons from one trap state to another distributed across different energies, is found to best represent the leakage evolution. The VRH mechanism, based on the theory developed by Mott and Hill, is illustrated in Figure 4b. The primary trap location is at A, with an energy *E_T_* and an exponentially distributed density of states (DOS). Electrons can hop from A to empty trap positions situated at B or C, within a distance of R from the primary trap, and within a range of energy surrounding *E_T_*.

**Figure 6:**
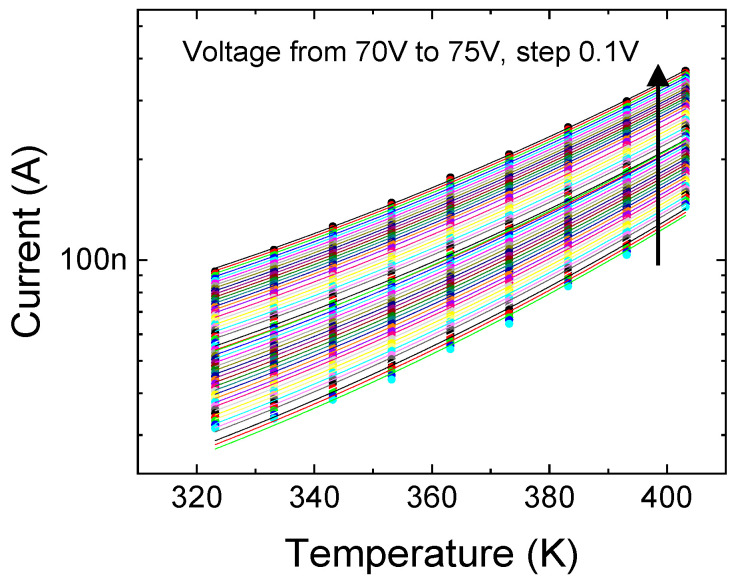
Modeling reverse leakage diode behavior using variable range hopping (VRH) from V= −70 V to −75 V (Region 2) for T = 50 °C (323 K) to 130 °C (403 K).

**Figure 7:**
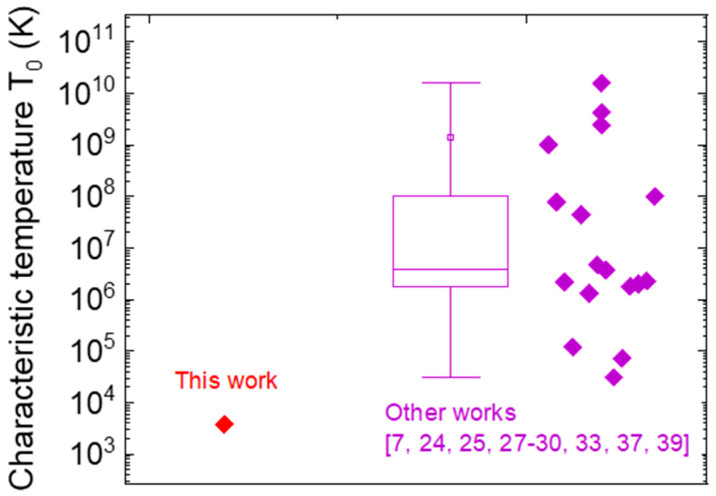
Comparison of the characteristic temperature (T_0_) obtained from different works on GaN-based systems [7,24,25,27,28,29,30,33,37,39].

The fit to measurements is presented in Figure 6 and modelled using the following relation with temperature *T* [7,35], valid for moderate to high electric fields, where $F^{2}$ represents the field contribution to strengthening the VRH conduction:

(3) IVRH=I0exp[−1.76(T0T)14+CVRH(T0T)34F2],

*I*_0_ modifies the trap emission rate into current, C_VRH_ represents a grouped constant, which can be written as CVRH=4.626×10−3×(qaU)2 [7], where *q* is the elementary charge, while *a* and *U* are constants related to the physical properties of the trap states. U indicates the characteristic energy of the DOS, and a represents the localization radius of the wave function corresponding to the trapped electron. It can be estimated to the effective Bohr radius of the bound electron and lies within the range of 1 nm to 10 nm [7,27,30,36]. *T*_0_, the characteristic temperature, can be written as T0=18(kBDTa3) [7,35,37,38]. It is inversely proportional to the trap DOS *D_T_* (volume^−1^ energy^−1^) at the primary (see A in Figure 4b) trap energy and can vary depending on a. A summary of different *T*_0_ values reported in the literature [7,24,25,27,28,29,30,33,37,39] is provided in Figure 7 for reference, showing consistent results for the values extrapolated in this paper.

Based on these results, we conclude that a substantial reduction in leakage current and in its temperature sensitivity can be obtained through the reduction of the density of defects within the drift region. Specific attention needs to be focused on the residual carbon concentration, considering its contribution to the low voltage regime.

### 3.2. TCAD Simulations of Diode Breakdown

**Figure 8:**
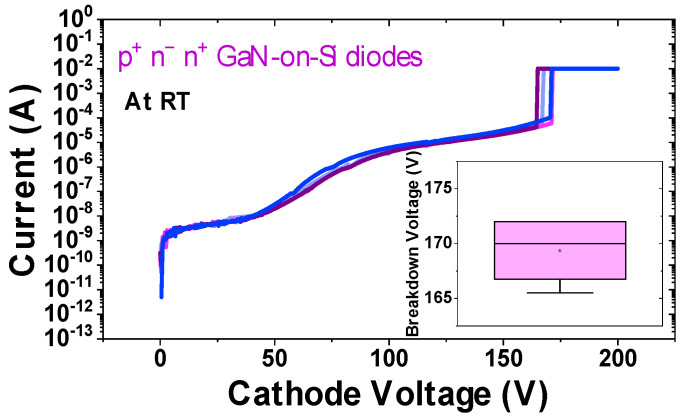
Reverse bias I–V diode characteristics until failure on four diodes. (inset) Mean breakdown voltage is 170 V.

To obtain an estimate of the breakdown voltage of the test structures, several samples were subjected to reverse bias sweeps until failure at room temperature, as illustrated in Figure 8. Very little dispersion was observed within the diode characteristics, and the mean reverse breakdown voltage was found to be around 170 V (inset of Figure 8).

**Figure 9:**
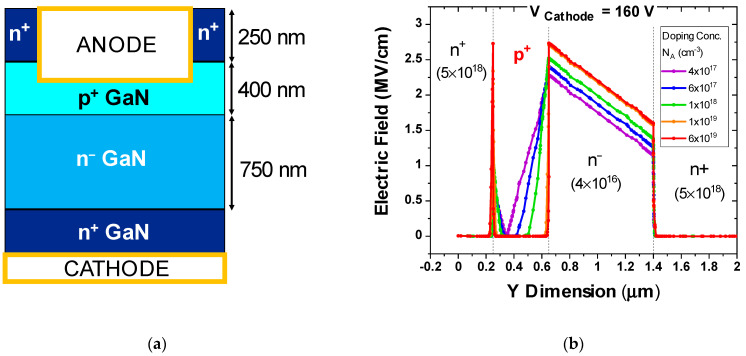
(**a**) Schematic of the simulated structure. (**b**) Electric field profile along the simulated vertical p^+^n diode for N_A_ = 4 × 10^17^ cm^−3^, 6 × 10^17^ cm^–3^, 1 × 10^18^ cm^−3^, 1 × 10^19^ cm^−3^, and 6 × 10^19^ cm^−3^.

The 2D-TCAD simulations, based on the drift–diffusion model for carrier transport, were employed to build a representative model of the measured devices, using the Sentaurus tool from Synopsys [40]. To investigate the nature of breakdown, a simplified fully vertical (n^+^-p^+^-n^−^-n^+^) diode structure (see Figure 9a) was used. The anode and cathode are defined as modified ohmic contacts to improve accuracy around the p–n junction; it does not impose the charge neutrality condition at vertices within the charged depletion regions [40]. In addition to suitable mobility and recombination models, the gate-dependent strain polarization model, especially suited to GaN devices, was activated. Since Mg has a relatively high ionization energy of 0.16 eV [41,42], Mg acceptors are not completely ionized at room temperature. Thus, to correctly model the p-doping levels and reproduce the breakdown voltage, the incomplete ionization model in Sentaurus was used, which is physically more accurate to model Mg doping. The effective doping concentration was computed internally based on ionization probability, derived from the ionization energy of the doping species.

Figure 9b presents the electric field evolution along the simulated structure as a function of the chosen p-doping level, for a cathode voltage of 160 V, i.e., just below the measured breakdown voltage.

**Figure 10:**
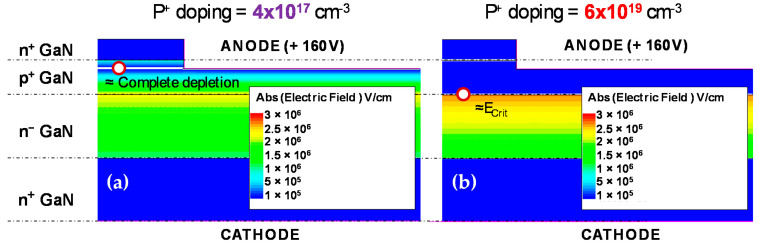
Two failure processes are identified from electric field profiles: (**a**) For lowly doped samples, punch-through occurs, due to the full depletion of the p-GaN, and (**b**) for high p-GaN, doping breakdown corresponds to the voltage for which the peak E-field at the p^+^n junction reaches the critical field of GaN.

For high p-doping, such as for N_A_ = 6 × 10^19^ cm^−3^ (see Figure 10b), we observed the peak electric field at the p^+^ to n^−^ interface approaching the critical field value for GaN (3.3 MV/cm [43]). The applied potential dropped almost entirely across the n-drift region, with little to negligible depletion observed within the p-GaN layer. As such, we can expect breakdown to occur when the peak electric field crosses E_Crit_.

**Figure 11:**
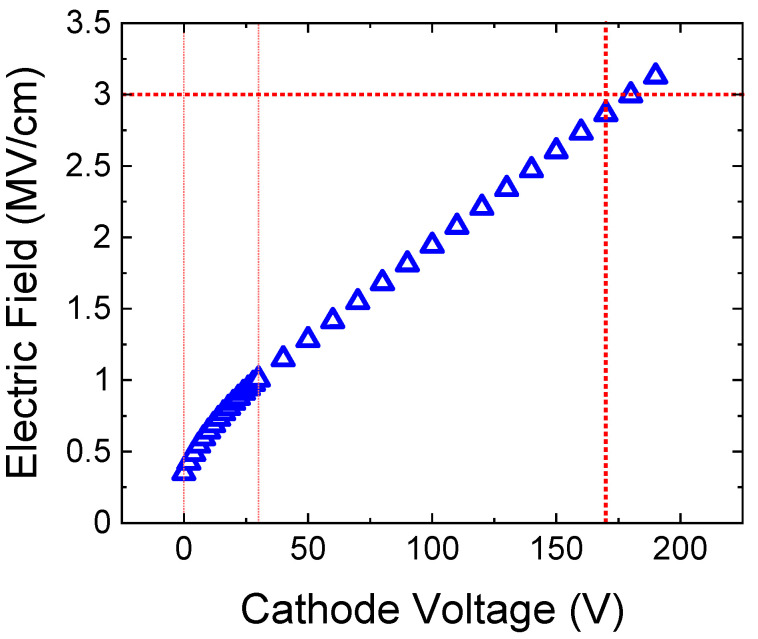
Electric field profile versus voltage for N_A_ = 6 × 10^19^ cm*^−^*^3^, corresponding to the devices under test, illustrating a field value ≈3 MV/cm at 170 V.

For the devices under test, the p-doping level was N_A_ = 6 × 10^19^ cm^−3^, which indicates breakdown triggered by peak electric fields >3 MV/cm at the p^+^n junction, as can be seen from the evolution of the electric field with increasing voltage in Figure 11.

The field values obtained in the range of 0 V–30 V were used in Figure 3b to verify the $\Delta E_{A}\;\propto \sqrt{F}$ dependence. It can be expected that by decreasing the p-doping within a certain range, we can reduce the peak electric field (see Figure 10b for N_A_ = 1 × 10^18^ cm^−3^), pushing V_BR_ to higher voltages >200 V. Simulations indicated that reducing the p-doping significantly introduces a different constraint. For example, for N_A_ = 6 × 10^17^ cm^−3^, we observed a much wider depletion of the p-GaN region. If the p-doping levels are too low (N_A_ = 4 × 10^17^ cm^−3^ in Figure 9b and Figure 10a), complete depletion of the pGaN layer can occur, thus leading to punch-through even before the peak electric field reaches E_Crit_. This effect may be further worsened under real conditions by the presence of hydrogen during the epi-growth process [44], which would reduce the effective concentration of Mg and accelerate the punch-through.

**Figure 12:**
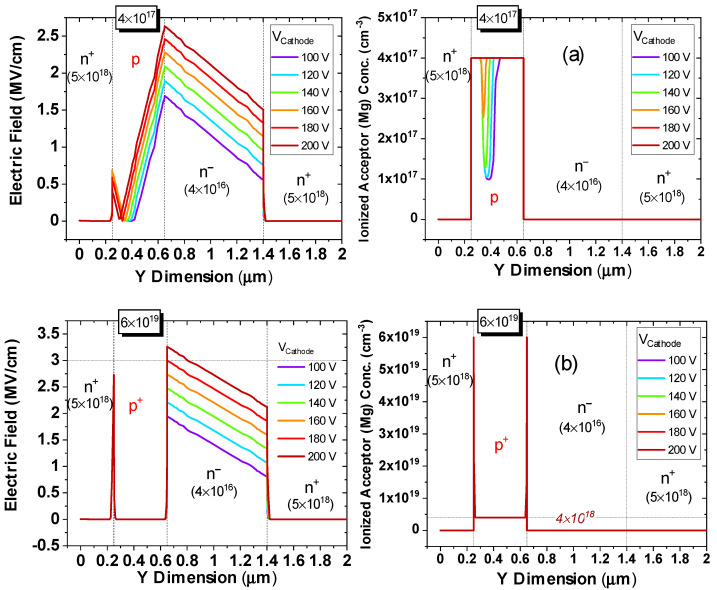
Electric field profile and ionized Mg acceptor concentration for simulations at different voltages with the incomplete ionization model: (**a**) low p-doping, N_A_ = 4 × 10^17^ cm*^−^*^3^, (**b**) high p-doping, N_A_ = 6 × 10^19^ cm*^−^*^3^. A trade-off exists on the value of p-doping: it must be sufficiently low to reduce the peak field, and sufficiently high to avoid punch-through.

Figure 12 illustrates the evolution of electric field and ionized acceptor concentration with cathode voltage, for low (N_A_ = 4 × 10^17^ cm*^−^*^3^) and high p-doping (N_A_ = 6 × 10^19^ cm*^−^*^3^) cases.

First, from Figure 12b we note that the simulations accurately reproduced the breakdown voltage of 180 V, for the samples under analysis, that had a doping level equal to N_A_ = 6 × 10^19^ cm*^−^*^3^. Second, we note that for the low N_A_ case (Figure 12a), the ionized Mg concentration followed the progressive depletion of the p-GaN layer with higher voltages, with complete ionization at voltages higher than the punch-through voltage.

Finally, for high N_A_, the base ionization level was constant at 6% (=4 × 10^18^ cm*^−^*^3^) of the defined N_A_ for most of the p^+^ GaN layer, except at the pn junctions, where the Mg acceptors were almost fully ionized.

Our conclusion on this part is that a trade-off exists on the value of p-doping, which must be sufficiently low to reduce the peak field, and sufficiently high to avoid punch-through.

## 4. Conclusions

In summary, we presented a detailed analysis of the leakage and breakdown limits of semi-vertical GaN-on-Si test structures. The results of the analysis indicated that thermionic carrier emissions from a trap state of 0.85 eV dominates leakage at low voltage, while variable range hopping is observable at high voltage.

TCAD simulation of the p^+^n diodes is employed to reproduce the breakdown voltage of semi vertical GaN on Si diodes. For the measured test structures with N_A_ = 6 × 10^19^ cm^−3^, breakdown is estimated to correspond to the voltage for which the peak E-field at the p^+^n junction reaches the critical field of GaN, and simulation can effectively reproduce the experimental results. For lowly doped samples, punch-through occurs, due to the full depletion of the p-GaN, as demonstrated by simulations. In conclusion, the doping of the p-GaN layer can strongly impact the breakdown voltage of the analyzed structures, and a trade-off between the occurrence of punch-through and junction breakdown needs to be considered to optimize device robustness.

## Figures and Tables

**Table 1:** Structural properties of the vertical GaN-on-Si diodes under test.

Layer (GaN)	Doping (cm^−3^)	Thickness (nm)
n^+^	5 × 10^18^	250
p^+^ body	6 × 10^19^	400
n^−^ drift	4 × 10^16^	750
